# The rate of orthokeratology lens use and associated factors in 33,280 children and adolescents with myopia: a cross-sectional study from Shanghai

**DOI:** 10.1038/s41433-023-02503-1

**Published:** 2023-04-12

**Authors:** Wenchen Zhao, Jingjing Wang, Jun Chen, Hui Xie, Jinliuxing Yang, Kun Liu, Xiangui He, Xun Xu

**Affiliations:** 1 Shanghai Eye Disease Prevention and Treatment Center, Shanghai Vision Health Center & Shanghai Children Myopia Institute, Shanghai Eye Hospital, Shanghai, 200040 China; 2 National Clinical Research Center for Eye Diseases, Shanghai Key Laboratory of Ocular Fundus Diseases, Shanghai General Hospital, Shanghai Jiao Tong University, Shanghai, 200080 China

**Keywords:** Epidemiology, Therapeutics

## Abstract

**Objectives:**

To investigate the rate of orthokeratology lens (ortho-k lens) use and its associated factors in children and adolescents with myopia.

**Methods:**

Cross-sectional study. Children from 104 primary and middle schools in Shanghai were enrolled by cluster sampling. Ophthalmic examinations were conducted and information was obtained using questionnaires for associated factors analysis.

**Results:**

A total of 72,920 children and adolescents were included, among which 32,259 were the potential population for ortho-k lens use. A total of 1021 participants used ortho-k lenses, equating to a use rate of 1.4% in the total population and 3.1% in the potential population. Age (OR 0.91, 95% CI: 0.88–0.95, *p* < 0.001), BMI (≥95th percentile: OR 0.48, 95% CI: 0.35–0.66, *p* < 0.001), age at initiation of refractive correction (≤12 years: OR 1.75, 95% CI: 1.31–2.33, *p* < 0.001), and parental myopia (either: OR 2.09, 95% CI: 1.58–2.75, *p* < 0.001; both: OR 3.94, 95% CI: 3.04–5.11, *p* < 0.001) were independently associated with ortho-k lens use. Of the ortho-k lenses users, 12.4% had a logMAR CVA of ≥0.3. A correction target (SE) of ≤−3.0 D (OR 2.05, 95% CI: 1.38–3.05, *p* < 0.001) and a sleeping duration of ≤6 h (OR 4.19, 95% CI: 2.03–8.64, *p* < 0.001) were factors independently associated with CVA ≥ 0.3.

**Conclusions:**

A certain proportion of children and adolescents in Shanghai chose to wear ortho-k lenses, related to the situation of parents and children themselves. Health education and follow-ups should be strengthened to ensure orthokeratology application quality.

## Introduction

The worldwide prevalence of myopia has risen sharply over the years, with the prevalence in certain regions, such as China, Japan and South Korea, reaching ≥80% and the prevalence of high myopia >10% [1–5]. Myopia progression is usually accompanied by axial length elongation [6, 7]. The stretching of eyeball wall in high myopia can induce various complications, which may lead to visual impairment and blindness [8–11]. Thus, effective measures should be taken to control myopia progression once it occurs.

Meta-analyses have indicated that orthokeratology lenses (ortho-k lenses) can reduce axial growth by 0.15 mm/year or 0.26–0.27 mm in 2 years in individuals with low and moderate myopia [12–16]. The process of corneal reshaping can decrease peripheral hyperopic defocus to increases peripheral myopic defocus and reduce stimuli for axial elongation, which is a possible mechanism for delaying the progression of myopia [17]. Ortho-k lenses are worn to flatten the cornea overnight, and wearers consequently possess good unaided vision during the daytime [18]. With ortho-k lenses becoming a widely used optical control intervention, the efficacy and safety of ortho-k lenses have been well studied; however, few studies have examined the use rate and other associated factors of ortho-k lens use.

In light of this research gap, our study was designed to explore the rate of ortho-k lens use and its associated factors in a large paediatric population aged 6–18 years, as well as the distribution of daytime corrected visual acuity (CVA) after one night of wearing ortho-k lenses.

## Materials and methods

### Study design and population

As a sub-project of the Shanghai Child and Adolescent Large Eye Study, some detailed methods of the study have previously been reported [19]. In this cross-sectional study, primary and secondary schools in Shanghai were selected by cluster sampling in 2019. A total of 104 schools were included, including 36 primary schools, 45 junior high schools, and 43 senior high schools. Written informed consent was obtained from the parents or guardians of the participants, and verbal consent was obtained from the participants. The tenets of the Declaration of Helsinki were followed, and the Institutional Review Board of Shanghai General Hospital, Shanghai Jiao Tong University, approved the study (ID: 2015KY149). Related supporting information has been provided in the methodology article published previously [19]. Basic information, such as height and weight, was collected from all participants, ophthalmic examinations were performed, and the participants were asked to complete a questionnaire.

The exclusion criteria of the study were as follows: (1) eye diseases other than refractive errors; (2) a history of eye trauma or eye surgery; (3) failure to complete the questionnaire or to undergo ophthalmic examinations; (4) lack of refractive information or conjoint use of any other optical correction except single vision spectacles by ortho-k lens users. Primary outcome measures were the rate of ortho-k lens use and its associated factors. Secondary outcome measures were the rate and associated factors of users with unsatisfactory daytime vision.

### Ophthalmic examinations

Ophthalmic examinations were performed for all children by specialized and experienced ophthalmologists or optometrists from local community hospitals. An international standard logarithmic visual acuity chart was used to measure unaided vision for all children and CVA for children wearing spectacles or lenses. Visual acuity measurements were recorded as the logarithm of the minimum angle of resolution (logMAR). Autorefraction was performed with an autorefractor (model KR-8900; Topcon, Tokyo, Japan) without cycloplegia. Five readings were obtained and averaged, all of which had to be less than 0.25 dioptre (D) apart. The spherical equivalent (SE) was calculated as the sum of the spherical power plus half of the cylindrical power. All of the examinations were carried out on school days and completed between 8 a.m. and 3 p.m.

### Questionnaires

Children’s parents or guardians, together with children themselves, were required to complete an online questionnaire prior to the ophthalmic examination. The questionnaire assessed the chosen correction method at the time; the power of spectacles or lenses, including ortho-k lenses; basic information; premature birth; the refractive status of the parents; age at the start of refractive correction and the sleeping duration on school days. In the questionnaire, the chosen correction method by the time included the following options: single-vision spectacles, defocus spectacles, soft contact lenses, ortho-k lenses, and others.

### Definition and evaluation criteria

In this study, the unaided daytime vision after one night’s use of ortho-k lenses was regarded as the CVA. An unsatisfactory CVA was defined as a CVA of ≥0.3 considering that this is the boundary visual acuity of mild visual impairment based on standard WHO definition [20]. The target of correction of ortho-k lenses (regarded as SE [D]) was defined as the sum of the spherical power plus half of the cylindrical power of the lens. Low myopia was defined as an SE of >−3.0 D, and moderate to high myopia was defined as an SE of ≤−3.0 D.

According to the range of applications of ortho-k lenses registered in the National Medical Products Administration, the spherical degree of an ortho-k lens wearer should be between 0 D and −6.0 D, while the cylindrical degree should be ≤2.0 D. In clinical practice, ortho-k lenses are commonly used within a correction range of −5.0 D ≤ SE ≤ − 1.0 D; therefore, in this study, the potential population was defined as the potential clinical user of ortho-k lens, with −5.0 D ≤ SE ≤ − 1.0 D and cylindrical degree ≤2.0 D.

Body mass index (BMI) was calculated as weight (kg) divided by height squared (m^2^). Underweight, healthy, overweight, and obesity were categorized according to recent published research [21, 22].

### Statistical analysis

Weight and BMI values less than the 1st percentile and greater than the 99th percentile were removed. Due to the high correlation between both eyes in the study, only the refractive and visual acuity data of the right eye were included in the analysis (Pearson’s correlation coefficient = 0.908, *p* < 0.001). The ortho-k lens wearing rates in the total population and in the potential population were calculated. After verifying normal distribution and homogeneity of variance, parametric variables were compared using an independent-samples *t*-test and are expressed as mean ± standard deviation (SD). Dichotomous variables were compared using the χ2 test according to grouping and are expressed as *N* (%). Otherwise, *u*-test or other methods were used for comparison. The selection of variables included in the regression model considered both the results of univariate analysis and the clinical significance of variables. A *P* < 0.05 was considered statistically significant.

## Results

### General characteristics of participants

**Fig. 1:**
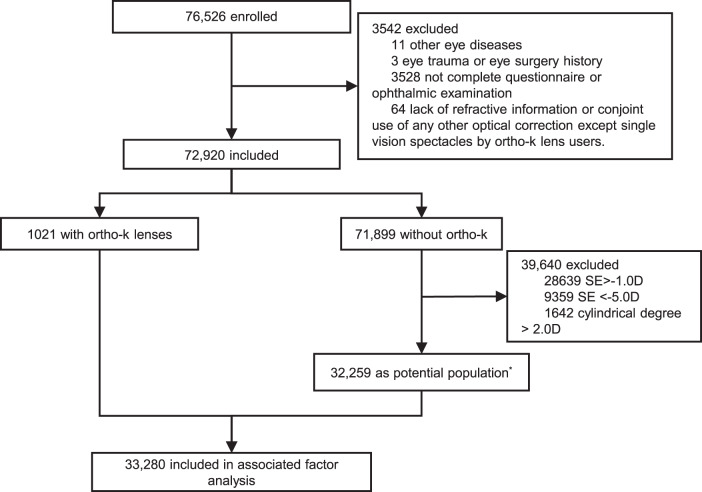
The inclusion criteria and screening process of this study. *The potential population of ortho-k lens use was defined as −5.0 D ≤ SE ≤ − 1.0 D and a cylindrical degree of ≤2.0 D for non-users.

A total of 76,526 children and adolescents from 104 primary and secondary schools were enrolled. A total of 3542 met the criteria for exclusion. Finally, 72,920 children and adolescents (37,170 boys and 35,750 girls) were included in the analysis. The participants ranged in age from 6 to 18 years (mean age, 11.96 ± 3.52 years). Of these, 1021 wore ortho-k lenses, while 71,899 did not. The selected potential population of non-wearers comprised 32,259 participants (15,659 boys and 16,600 girls), with a mean age of 13.04 ± 2.95 years, while the 1021 wearers (417 boys and 604 girls) had a mean age of 12.38 ± 2.57 years. The detailed screening process is shown in Fig. 1. The basic information of the participants is shown in Table 1. Age, sex, height, and weight of children from enrolled schools and those from non-enrolled schools were compared and that our sample was representative.

**Table 1:** Basic information of enrolled participants.

Age, years	Total population, *n*	Potential population, *n*^a^	Ortho-k lens wearers, *n*	Wearing rate in total population, %^b^	Wearing rate in potential population, %^c^
6^d^	4558	290	1	0.0	0.3
7	6059	963	10	0.2	1.0
8	5534	1376	46	0.8	3.2
9	5245	1882	85	1.6	4.3
10	4796	2127	131	2.7	5.8
11	6752	3516	131	1.9	3.6
12	6818	3829	154	2.3	3.9
13	5424	3149	126	2.3	3.9
14	4827	2765	79	1.6	2.8
15	7384	4079	115	1.6	2.7
16	7502	4074	77	1.0	1.9
17	6780	3583	60	0.9	1.7
18^e^	1241	626	6	0.5	1.0
Total	72,920	32,259	1021	1.4	3.1

^a^The potential population of ortho-k lens use was defined as −5.0 D ≤ SE ≤ − 1.0 D and a cylindrical degree of ≤2.0 D for non-users.

^b^Wearing rate in the total population = $\frac{{{{{{{{{\mathrm{Ortho - k}}}}}}}}\;{{{{{{{\mathrm{lens}}}}}}}}\;{{{{{{{\mathrm{wearers}}}}}}}}}}{{{{{{{{{\mathrm{Total}}}}}}}}\;{{{{{{{\mathrm{population}}}}}}}}}}$ × 100%

^c^Wearing rate in the potential population = $\frac{{{{{{{{{\mathrm{Ortho - k}}}}}}}}\;{{{{{{{\mathrm{lens}}}}}}}}\;{{{{{{{\mathrm{wearers}}}}}}}}}}{{{{{{{{{\mathrm{Ortho - k}}}}}}}}\;{{{{{{{\mathrm{lens}}}}}}}}\;{{{{{{{\mathrm{wearers + Potential}}}}}}}}\;{{{{{{{\mathrm{population}}}}}}}}}}$ × 100%

^d^A total of 4 5-year-old were included in the 6-year-old age group, in which there were no ortho-k lens users.

^e^A total of 161 19-year-olds and 13 20-year-olds were included in the 18-year-old age group, in which there were no ortho-k lens users.

### Ortho-k lens wearing rate

**Fig. 2:**
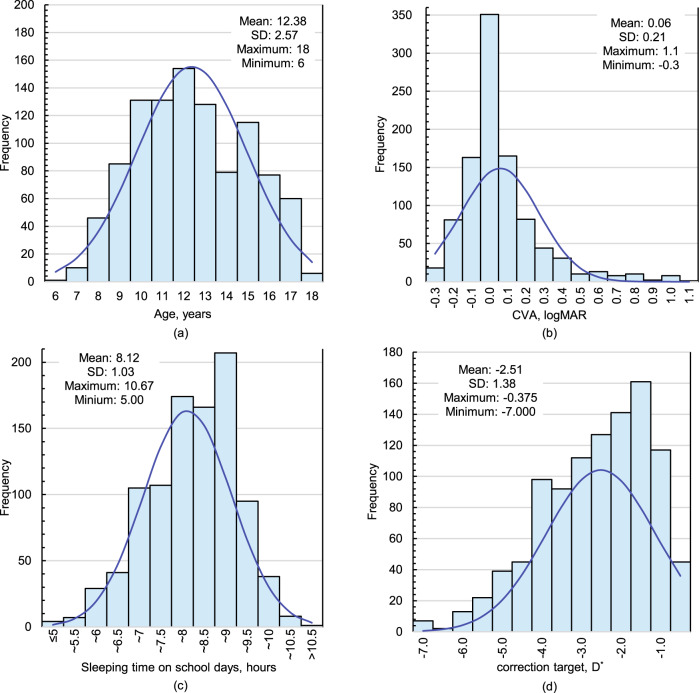
Basic information and wearing conditions of ortho-k lens users. The distribution, range and average of age (**a**), CVA (**b**), sleeping time on school days (**c**), and correction target (**d**) of ortho-k lens users. CVA corrected vision acuity, D dioptre. *The correction target of ortho-k lenses was defined as the sum of the spherical power plus half of the cylindrical power of the lens.

The distribution of age and correction target in ortho-k lens users is shown in Fig. 2. The children who wore ortho-k lenses were mainly concentrated in the 10–13-year age group, which comprised 53.2% of the total wearers, with the majority (15.1%) being 12 years of age. Up to 53.0% of ortho-k lens users had a correction target (SE) of between −3.0 D and −1.0 D.

As shown in Table 1, the ortho-k lens wearing rate was 1.4% in the total population and 3.1% in the potential population. The ortho-k lens wearing was concentrated in certain age groups, with the highest rate appearing at the age of 10 years (2.7% in the total cohort and 5.8% in the potential population) and a gradual decrease in wearing rate in children older or younger than 10 years. Children aged 9–13 years showed a higher wearing rate than other age groups in the potential population (*χ*^2^ = 88.25, *p* < 0.001, *χ*^2^ test).

### Factors associated with ortho-k lens use

The comparisons between ortho-k lens users and non-users are shown in Table 2. The differences in sex, age, height, weight, BMI, academic stage, parental myopia, and age at initiation of refractive correction between ortho-k lens users and non-users were statistically significant (*p* < 0.001 for all). No significant differences were observed between the two groups in premature delivery (*p* = 0.697), and sleeping duration on school days (*p* = 0.320).

**Table 2:** Comparisons between ortho-k lens users and non-users.

		Total (*n* = 33344)		Ortho-k lens users (*n* = 1021)		Ortho-k lens non-users (*n* = 32259)		*p* value
		Mean ± SD^a^	Range	Mean ± SD^a^	Range	Mean ± SD^a^	Range	
Boys, *n* (%)		16076 (48.3)	–	417 (40.8)	–	15659 (48.5)	–	<0.001^b^*
Age, years		13.02 ± 2.94	6~18	12.38 ± 2.57	6~18	13.04 ± 2.95	6~18	<0.001^c^*
Height, cm		158.00 ± 14.27	109.2~197.0	156.80 ± 12.83	119.5~192.8	158.03 ± 14.31	109.2~197.0	0.005^c^*
Weight, kg^d^		51.59 ± 15.46	16.5~116.3	48.44 ± 13.36	21.0~103.0	51.69 ± 15.51	16.5~116.3	<0.001^c^*
BMI, kg/m^2d^		20.20 ± 3.82	13.27~33.22	19.31 ± 3.24	13.31~32.95	20.22 ± 3.84	13.27~33.22	<0.001^c^*
Premature delivery, *n* (%)		1553 (4.7)	–	50 (5.0)	–	1503 (4.7)	-	0.697^b^
Age at initiation of refractive correction, years		10.90 ± 2.53	0~18	9.54 ± 2.32	1~17	10.96 ± 2.52	0~20	<0.001^c^*
Sleeping time on school days, hours		8.09 ± 1.07	4.85~12.08	8.12 ± 1.02	5.00~10.67	8.09 ± 1.16	4.85~12.08	0.320^c^
Parental myopia, *n* (%)	Neither	12240 (40.4)	–	155 (16.1)	–	12085 (41.2)	–	<0.001^b^*
	Either	9533 (31.5)	–	278 (28.8	–	9255 (31.6)	–	
	Both	8515 (28.1)	–	531 (55.1)	–	7984 (27.2)	–	
Academic stage, *n* (%)	Primary school	7195 (21.6)	–	285 (27.9)	–	6910 (21.4)	–	<0.001^b^*
	Junior high school	13834 (41.6)	–	483 (47.3)	–	13351 (41.4)	–	
	Senior high school	12251 (36.8)	–	253 (24.8)	–	11998 (37.2)	–	

*BMI* body mass index.

*P < 0.05 was considered statistically significant.

^a^Except where noted otherwise.

^b^Independent-samples *t*-test was used to identify statistical significance.

^c^χ2 test was used to identify statistical significance.

^d^Weight and BMI values less than the 1st percentile and greater than the 99th percentile were removed.

**Fig. 3:**
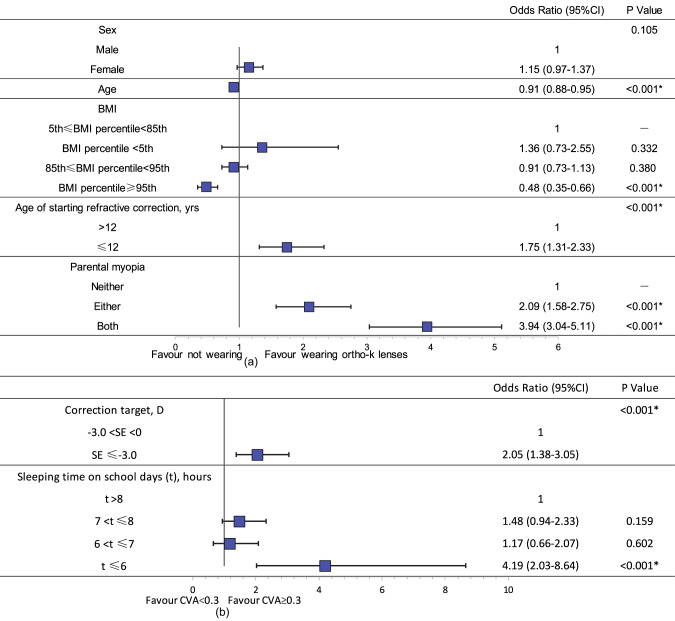
Factors independently associated with ortho-k lens use and unsatisfactory CVA. Forest plots of binary logistic regression model and its results with ortho-k lens use (**a**) and unsatisfactory CVA (**b**) as dependent variables respectively. The selection of variables considered both statistical results and clinical significance. BMI body mass index, D dioptre, CVA corrected visual acuity. *P < 0.05 was considered statistically significant. ^a^The correction target of ortho-k lenses was defined as the sum of the spherical power plus half of the cylindrical power of the lens.

Considering the results of univariate analysis and the clinical significance of variables, sex, age, BMI, age at initiation of refractive correction, and parental myopia were included in the binary logistic regression model with weather wearing ortho-k lens or not as dependent variable [Fig. 3a]. It is indicated that starting refractive correction at ≤12 years of age (odds ratio [OR] 1.75, 95% confidence interval [CI] 1.31–2.33, *p* < 0.001) and parental myopia (either: OR 2.09, 95% CI: 1.58–2.75, *p* < 0.001; both: OR 3.94, 95% CI: 3.04–5.11, *p* < 0.001) were positively associated with ortho-k lens use, while age (OR 0.91, 95% CI: 0.88–0.95, *p* < 0.001) and obesity (OR 0.48, 95% CI: 0.35–0.66, *p* < 0.001) were negatively associated with ortho-k lens use. Sex was not independently related to ortho-k lens use (*p* = 0.105).

### Unsatisfactory CVA in ortho-k lens users and associated factors

The distribution of daytime CVA in ortho-k lens users is shown in Fig. 2. Of the 1021 ortho-k lens users, 127 participants (12.4%) had a CVA of ≥0.3 in the right eye. Multiple regression analysis of factors associated with unsatisfactory CVA was performed and the results indicated that correction target (*p* < 0.001) and sleeping time on school days (*p* = 0.008) were independently associated with a CVA of ≥0.3, while no significant differences were observed in other factors (*p* > 0.05) (Supplementary Table 1). In the binary logistic regression model with unsatisfactory CVA as dependent variable [Fig. 3b], SE ≤ − 3.0 D (OR 2.05, 95% CI: 1.38–3.05, *p* < 0.001) and a sleeping duration of ≤6 h on school days (OR 4.19, 95% CI: 2.03–8.64, *p* < 0.001) were independently associated with an unsatisfactory CVA. As shown in Fig. 2, 4.1% of children who wore ortho-k lenses slept for no more than 6 h.

## Discussion

To our knowledge, this is the first report to analyse the use rate of ortho-k lenses in school-aged children and adolescents. Our study found that a certain proportion of children and adolescents chose to wear ortho-k lenses, some of which had unsatisfactory daytime vision. Age, BMI, age at initiation of refractive correction, and parental myopia were independently associated with ortho-k lens use.

In our study, ortho-k lenses had a use rate of 1.4% in the total cohort and 3.1% in children with myopia. In contact lens users, Morgan et al. reported an ortho-k lens use rate of 1.40% for people of all ages in several countries including China from 2004 to 2017 [23]. These results indicate a relatively low ortho-k lens use rate including in the myopic population. Optometry of ortho-k lens and its follow-ups require the support of experienced clinical doctors, adequate medical conditions, and good adherence of ortho-k lens users [24]. Moreover, adverse effects have been reported, which may be related to corneal hypoxia caused by use during sleep [18, 25, 26]. The features mentioned above may be some of the reasons why ortho-k lenses are not widely used.

Age and the age at initiation of refractive correction are independently associated with ortho-k lens use. In our study, junior high school children comprised the largest proportion of ortho-k lens users, followed by primary school students and senior high school children. This conclusion is similar to the results of Morgan et al., who reported that a high proportion of children aged 13–17 years and 6–12 years used ortho-k lenses [23]. Considering that ortho-k lenses are usually worn by children over the age of 8 years and the onset of myopia mostly appears at 9–10 years of age, our results are consistent with the clinical situation. As reported, an early age of myopia onset suggests a high risk of high myopia, and the likelihood of progressing to high myopia is high in children diagnosed with myopia before the age of 12 years [3, 27, 28]. Thus, it is gratifying to see that children who start refractive correction early are more likely to use ortho-k lenses. In addition, since the progression of childhood myopia slows with age [29–31], younger children may also benefit more from ortho-k lenses for myopia control than older children [32–34].

Parental myopia was independently associated with the choice to use ortho-k lenses (either: OR 2.09; both: OR 3.94). The understanding and attitude of parents toward myopia may influence the methods used to correct myopia, as well as the treatment compliance of children. Previous studies have found that parents adopt a proactive role when deciding on myopia treatment [24, 35], and parents with myopia may pay more attention to myopia development and control in their children than emmetropic parents [36]. Studies have also reported that the majority of parents obtain information on ortho-k through word of mouth or from ophthalmologists [32, 37], which may suggest a lack of public knowledge about myopia and its available treatments.

Our study found that children with obesity were less likely to wear ortho-k lenses than children with a healthy BMI, which was seldom mentioned in previous studies. Childhood obesity is often related to less physical activities, so children with a higher BMI may have less need to be lens-free during the day [38]. Previous studies have indicated that family income is negatively correlated with BMI in children. In addition, childhood temperament, especially lack of self-regulation and dissatisfaction with limitations, is considered to be an important predictor of obesity in children [39, 40]. Therefore, we speculate that children with a higher BMI are less likely to use ortho-k lenses due to less need, the economic conditions of their families and their lower self-management ability. Sex was not independently associated with ortho-k lens use, although girls were considered more likely to wear ortho-k lenses than boys in the univariable analysis. Sex and BMI are potential associated factors; thus, the findings presented here might provide some clues for future studies.

In our study, 12.4% of the participants who used ortho-k lenses had a CVA of ≥0.3, and ortho-k lens use was associated with a high lens degree and lack of sleep. Ortho-k lens changes an eye’s refractive state by flattening the cornea. Unsatisfactory vision is associated with higher correction target, possibly because the eye has limited deformability and is more likely to return to its original shape. Unsatisfactory vision potentially related to insufficient sleep time may also be related to insufficient deformation. Previous studies have rarely involved the influence of sleep time on the effect of ortho-k lens. Chen et al. studied the factors influencing the effect of ortho-k lens on the control of myopia progression and did not find significant correlation, but all children participants in the study had a sleeping time ≥8 h [41]. Previous studies have shown that daytime vision can reach 0.0 after ortho-k lens use and remain stable for longer than 6 months [34, 42–44]. The difference in daytime vision may be due to the fact that our study measured visual acuity in random examination, rather than under close follow-up in clinical trials. Chang et al. found that the compliance of children and their guardians decreased as the follow-up interval lengthened. Lack of compliance included not paying attention to lens hygiene and not examining or replacing lenses in a timely manner [35]. Therefore, ortho-k lenses should be used more specifically for children who meet the indications or with the need to take off glasses during daytime. And to ensure a better therapeutic effect and reduce the risk of adverse reactions, ophthalmologists should conduct more rigorous examination of indications and perform detailed examinations and inquiries during follow-ups in clinical practice, as well as adequately educate the children and their guardians. For children who are not suitable for ortho-k, other means of controlling myopia progression should be chosen instead, which is mentioned below. As for the unsatisfactory daytime vision potentially related to a short duration of sleep, some ophthalmologists suggest that children start to wear ortho-k lenses in the evening to make up the time, which might provide clues for future studies and clinical practices.

Several strategies have been introduced in China since 2016 to promote the early screening and correction of myopia in children. Visual acuity and autorefraction screening are performed at school in students’ annual physical examinations. Children who may be newly-developed myopia or have a rapid progression of myopia progress are directed to hospitals for detailed refractive examinations and treatments. Meanwhile, different myopia control measures have provided more choices for children with different circumstances. Low concentration atropine and orthokeratology have been included as appropriate technical in guidelines for myopia control in China, while newer measures such as peripheral defocus contact lens or ophthalmic lens are also available [45, 46]. Although the indications and selection of different measures remain to be summarized by the results of clinical trials, this is a step forward for myopia interventions toward routine treatment and wide application.

This study has several limitations that should be noted. First, the refractive status in this study was obtained without cycloplegia, which could result in overestimation of the potential population and correspondingly lead to a lower rate of ortho-k use. Actually, due to practical limitations, it is difficult for us to use cycloplegic optometry for all children in large-scale school examinations. Meta-analysis and study on children aged 4 to 15 showed that, non-cycloplegia refraction resulted in a mean SE difference of −0.60D compared to cycloplegia, with greater differences observed in younger participants and in eyes with more hyperopic refraction degree [47, 48]. Therefore, the influence of non-cycloplegia data on this study could be limited. Second, we lack information about the pharmaceutical agents they used, like atropine, which may affect our analysis of the results. Since atropine is not regularly used as a correction method and the usage and dosage information could be complicated to collect, we failed to collect the information of myopia interventions other than optical means. Nevertheless, we believe that the results of this study could still provide adequate information on the characteristic of ortho-k lens wearers. Third, since ortho-k lens could both control the progression of myopia and be a method of correction, the actual purpose of children wearing lenses may affect the results, which should be distinguished. It is a pity that due to the limitation of the method and sample size, this detail question was not included in our questionnaire, which is also one of limitations in this study and could be studied further. Fourth, this is a cross-sectional study; thus, no causal relationship can be concluded. The information on children and their families was collected using questionnaires, which carry a risk of recall bias. Fifth, our study lacks information about parental education, family economics, and other factors that may be associated with the choice to use ortho-k lenses and daytime vision. Sixth, the time of the examinations could not be unified; thus, the influence of examination time on CVA cannot be completely excluded. However, according to previous research results, visual acuity did not change significantly from 1 h to 10 h after removal of ortho-k lenses [44].

In conclusion, a certain proportion of children and adolescents in Shanghai chose to wear ortho-k lenses. Some of these children and adolescents exhibited a decline in daytime vision. Ortho-k lens is an effective method for myopia control, but for reasons related to safety and efficacy, examinations, health education and clinical follow-up of children who use these lenses must be strengthened. Public education is imperative so that the awareness of myopia control is increased in parents of young children and to help parents make informed decisions.

## Summary

### What was known before

- The efficacy and safety of ortho-k lenses have been well studied.

### What this study adds

- A certain proportion of children and adolescents chose to wear ortho-k lenses. Some of the users had unsatisfactory daytime vision. Age, BMI, age at initiation of refractive correction, and parental myopia were independently associated with ortho-k lens use.

### Supplementary information

Supplementary Table 1

## Data Availability

The data that support the findings of this study are available from the corresponding author upon reasonable request.
