# Supplementary material for: The rate of orthokeratology lens use and associated factors in 33,280 children and adolescents with myopia: a cross-sectional study from Shanghai
Source: Eye (Lond). 2023 Apr 12;37(15):3263–70. doi: 10.1038/s41433-023-02503-1 (PMC10564736; doi:10.1038/s41433-023-02503-1)
Supplement: Supplementary file 1 — Supplementary Table 1 [file 41433_2023_2503_MOESM1_ESM.doc]

|  | B | SE | Wald | f | P value | Odds Ratio (95% CI) | |
| --- | --- | --- | --- | --- | --- | --- | --- |
| Constant | -1.643 | 1.349 | 1.483 | 1 | 0.223 | 0.193 |  |
| Sex | -0.205 | 0.255 | 0.643 | 1 | 0.423 | 0.815 (0.494-1.344) |  |
| Age, years | -0.053 | 0.081 | 0.429 | 1 | 0.512 | 0.948 (0.809-1.111) |  |
| Age of starting refractive correction, years | 0.054 | 0.064 | 0.723 | 1 | 0.395 | 1.056 (0.932-1.197) |  |
| Parental Myopia |  |  |  |  |  |  |  |
| Neither |  |  |  | 2 | 0.295 |  |  |
| Either | 0.536 | 0.395 | 1.848 | 1 | 0.174 | 1.710 (0.789-3.706) |  |
| Both | 0.202 | 0.387 | 0.273 | 1 | 0.601 | 1.224 (0.573-2.615) |  |
| Correction target, D^a^ | -0.400 | 0.090 | 19.578 | 1 | <0.001* | 0.67 (0.56-0.80) |  |
| Sleeping time on school days (t), hours |  |  |  |  | 0.008* | 0.61 (0.42-0.88) |  |
| t >8 |  |  | 10.392 | 3 | 0.016* |  |  |
| 7 <t ≤8 | -1.194 | 0.511 | 5.467 | 1 | 0.019* | 0.303 (0.111-0.824) |  |
| 6 <t ≤7 | -1.090 | 0.513 | 4.518 | 1 | 0.034* | 0.336 (0.123-0.919) |  |
| t ≤6 | -1.808 | 0.591 | 9.349 | 1 | 0.002* | 0.164 (0.051-0.523) |  |

**Supplemental Table 1. Multiple logistic regression of influencing factors associated with unsatisfactory CVA.**

D: dioptre; CVA: corrected visual acuity.

^a^The correction target of ortho-k lenses was defined as the sum of the spherical power plus half of the cylindrical power of the lens.

* P < 0.05 was considered statistically significant.
